# Efficacy of Tranexamic Acid in Preventing Alveolar Osteitis in Post-extraction Sockets of First Premolars

**DOI:** 10.7759/cureus.51816

**Published:** 2024-01-07

**Authors:** Sharanika A Nagaja, Rubin S John, Murugesan Krishnan

**Affiliations:** 1 Oral and Maxillofacial Surgery, Saveetha Dental College and Hospitals, Saveetha Institute of Medical and Technical Sciences, Saveetha University, Chennai, IND

**Keywords:** post-operative pain, dental extraction, extraction sockets, topical tranexamic acid, alveolar osteitis

## Abstract

Objectives: The present study is a randomized trial for comparing the effectiveness of tranexamic acid as an antifibrinolytic agent in preventing alveolar osteitis in the post-extraction period in patients receiving orthodontic therapy that requires extraction.

Methodology: This research was carried out in the Department of Oral and Maxillofacial Surgery, Saveetha Dental College and Hospitals, Chennai, India. A total of 40 patients were considered subjects for the research. Patients undergoing orthodontic treatment referred to the Department of Oral and Maxillofacial Surgery for the therapeutic extractions of the first premolars were considered for this study. Randomization was done to split the population into study and control. After the atraumatic extraction of the first premolars under local anesthesia using 2% lignocaine with 1:80000 adrenaline, a tranexamic acid solution of 500 mg soaked gauze over the extraction sockets was used as the intervention in the study group, and plain gauze was used on the control group. Patients were asked to hold the gauze in place for one hour. Participants were reviewed after three days for the incidence of alveolar osteitis and pain severity and healing of the extraction sockets.

Results: The prevalence of Alveolitis sicca dolorosa was found to be 5% in the research group and 15% in the control group. Patients in the control group showed more pain than the patients in the research group. The period taken for healing ranged from 7 days to 10 days in the control group and 10 days to 12 days in the study group.

Conclusion: This study gives an edge that tranexamic acid can be used as a local hemostatic agent in preventing fibrinolysis of clots and preventing alveolar osteitis.

## Introduction

Dry socket or alveolar osteitis is one of the typical complications that are seen at the site of permanent teeth extraction [1]. Alveolar osteitis is post-operative pain that worsens between one and three days at the site of extraction and is also accompanied by a disintegration of blood clots partially or completely in the extraction socket which may or may not cause halitosis.

Alveolar osteitis is an inflammatory condition, where the bare bone is not covered partly or totally by a blood clot or epithelium. It can be seen inside or surrounding the alveolar socket for days in the post-extraction period [2]. Alveolitis lesion occurs in 0.5-5% of the extractions [3]. There is no sex predilection for the frequency of dry sockets following extraction [4]. Symptoms of alveolar osteitis can be seen as pain radiating to the ear and temporal areas, infrequent maxillary involvement in the ocular and frontal regions, halitosis, low-grade fever, exposed bone, inflammation of gingival margins, regional lymphadenopathy, and greyish discharge [5-8].

Tranexamic acid is a lysine amino acid derivative that is artificially synthesized, and an active trans-stereoisomer of aminomethyl cyclohexane carboxylic acid, which has an antifibrinolytic effect. It acts by blocking lysine binding sites on plasminogen molecules irreversibly. At easily attained serum concentrations, it inhibits plasminogen activation through competition. At higher serum levels, it also reduces preformed plasmin activity [9]. Tranexamic acid can be effectively used as a local anti-fibrinolytic agent to control bleeding after dental and minor oral surgical procedures. It can be used in controlling post-operative bleeding in dental patients on anticoagulant therapy and bleeding and clotting disorders [10].

This research was designed to determine the effectiveness of the course of action of tranexamic acid in the post-extraction sockets of first premolars for orthodontic treatment.

## Materials and methods

The research was conducted in the outpatient Department of Oral and Maxillofacial Dentistry at Saveetha Dental College and Hospitals in Chennai, India, from February 2023 to May 2023. It was a randomized controlled clinical trial. Ethical clearance was obtained from Saveetha Dental College - Institutional Human Ethical Committee (IHEC/SDC/OMFS-2203/23/288).

Population under investigation

Patients referred from the Department of Orthodontics to the outpatient Department of Oral and Maxillofacial Dentistry for the therapeutic removal of first premolars of the maxilla and mandible for the orthodontic treatment were considered for this study. Using random sequence allocation, the population was categorized into two equal groups in a 1:1 ratio. Group A: tranexamic acid-soaked gauze post-extraction (n=20) and group B: plain gauze past extraction (n=20).

Study participants

Patients included in this research were between the age groups 14 to 20 years, both genders requiring maxillary and mandibular first premolars therapeutic extraction for orthodontic treatment.

Inclusion criteria

Patients who agreed to provide informed consent for the extractions and those who accepted extraction, being willing to be part of the study, were included. Patients without a history of bleeding or clotting disorders, as well as those without any intraoral infection, were eligible for participation. However, patients with known allergies or contraindications for tranexamic acid were excluded from the study.

Scheduling

The consent pro forma contained the complete patient information. The treatment procedure to be performed was explained to the patients in their respective native languages.

Detailed protocol and conduction of study

The study process was explained to the participants, and their written and verbal consent was obtained in detail. The procedure was performed by a single oral and maxillofacial surgeon adhering to strict aseptic protocol, under local anesthesia. First premolars were extracted a-traumatically using premolar extraction forceps according to the orthodontist orders. In patients from the study group, 2 ml tranexamic acid soaked (100 mg/ml) in gauze (2.5 cm×2.5 cm) was placed at the site of extraction. In the patients from the control group plain gauze was used after the extraction. Patients were asked to hold the gauze in place for one hour.

Patients were provided with instructions after the procedure along with oral medications of amoxicillin 500 mg twice a day (BID), a combination of aceclofenac 100 mg and paracetamol 500 mg BID, and pantoprazole 40 mg once daily for three days.

Patients were recalled after three days post-procedure, a complete clinical examination was done to record the exposure of the bone (partly or wholly), the disintegration of blood clots from the sockets, and the pain was evaluated by applying visual analog scale (VAS) ranging from 1 to 10. Values ranging from 1 to 3 were considered mild pain, 4 to 6 were considered moderate pain, and 7 to 10 were considered severe pain.

## Results

This research considered 40 subjects, the age of the subjects varied between 14 and 20 years, divided into group A (n=20) and group B (n=20).

The prevalence of alveolitis was seen in three patients from the control group, which consisted of 15% of the subjects, of whom two patients had partial clot disintegration and one patient had complete clot disintegration with exposure of the entire bone socket. The frequency of alveolitis in the study group was observed in one patient which was 5% of the study population. There was partial clot disintegration with exposure of bony socket edges. The incidence of alveolar osteitis was observed in the mandible in both the study and control groups. A p-value of 0.0085 was observed which is significant.

The median of the VAS score was taken from both groups to calculate the difference. The study group had a median value of 5.6 and the control group had a mean value of 8.5 with a p-value of 0.0001 which showed a significant difference (Table 1).

**Table 1:** Comparison between study and control groups regarding the prevalence of alveolar osteitis, severity of pain, time taken for healing, and exposure of the sockets VAS: Visual analog scale

Criteria	Study group	Control group	p-value
Number of patients	20	20	-
Incidence of alveolar osteitis	1	3	0.0085 (Z-test)
Median of severity of pain on the VAS scale	5.6	8.5	0.0001 (Mann-Whitney test)
Clot disintegration			
Complete	0	2	
Partial	1	1	
Mean duration taken for healing after alveolar osteitis	8.50±0.50 days (7-10 days)	11±0.75 days (10-12 days)	0.0004 (T-test)
Exposure of the sockets			
Complete	0	1	
Partial	0	2	

A significant difference in the incidence of alveolar osteitis is seen between the control population and the study populations. In the study population, none of the patients had complete exposure to the sockets, this might been the reason for the faster healing and decreased duration of time in the study population in contrast to the control population. But, this needs further studies on larger scales and extensive follow-up.

## Discussion

The outcome of our research shows that the local administration of tranexamic acid significantly reduces the incidence of alveolitis in sockets after extraction of first premolars. It is been observed there is more incidence of alveolitis in the mandible than in the maxilla, and the use of tranexamic acid has decreased the incidence. This is a significant clinical observation, Because alveolalgia is a typical consequence after the tooth extraction, leading to considerable patient discomfort and delayed healing. By maintaining the integrity of the blood clot within the socket, tranexamic acid likely reduces the exposure of bone and inflammation, thereby preventing the development of alveolar osteitis. Alveolitis is the most frequent unwanted post-operative consequence during the post-extraction period. There are numerous methods suggested, like systemic antibiotics like penicillin, metronidazole, and topical antibiotics [6-8,11].

In an article by Birn et al., he explained that the etiology of alveolitis is caused by elevated regional fibrinolysis which causes the clot to disintegrate. The disintegration of a clot is caused by the activation of the plasminogen pathway, the result of plasminogen pathway activation which can be achieved via direct (physiologic) or indirect (non-physiologic) chemical substances. After the extraction of the tooth, direct activators are released. Bacteria generate indirect activators. Because the early ingression of plasminogen into the clot inhibits the activity of plasmin, the fibrinolytic activity is local [12-17].

An article by Kolokythas et al. has described the contributing factors causing alveolar osteitis. These include surgical trauma and difficulty of the procedure, inexperienced surgeons, third molars, systemic health, oral contraceptives, gender, smoking, the disintegration of the blood clot, bacterial infection, extensive irrigation or curettage of the alveolus, age, local anesthetic agents with vasoconstrictor, saliva, bone/root fragments remaining in the wound, flap design/use of sutures [3].

The findings of this study have important clinical implications. Tranexamic acid could be considered as a preventive measure in patients at an increased rate of encountering alveolar osteitis, such as those with a previous incidence of alveolitis or tobacco use. Its ease of application, low cost, and lack of serious adverse events make it an attractive option for improving post-extraction outcomes. However, there are several avenues for future research that should be explored. Long-term follow-up studies are needed to determine the duration of tranexamic acid's protective effect and whether it has any impact on the long-term bone healing process. Additionally, investigating the optimal concentration and application method of tranexamic acid would help refine its clinical application [18-23].

Our current study is limited to individuals without any health complications and does not consider body weight. Further research has to be done to study the effect of tranexamic acid in patients with bleeding and clotting disorders. Proper dosage can be tailored according to the patient’s body weight, health conditions, and gender.

## Conclusions

Finally, our research reveals that the local application of tranexamic acid significantly reduces the prevalence of dry sockets, improves wound healing, and decreases post-operative pain. Tranexamic acid's anti-fibrinolytic properties likely contribute to its preventive effects by maintaining the stability of the blood clot. These findings provide a promising avenue for enhancing post-extraction recovery and warrant further research to explore its broader applications in dental surgery. More research with bigger sample numbers and extensive periods of follow-up is needed to corroborate these findings and explore the broader applicability of tranexamic acid in dental surgery.
